# Therapeutic Alliance in Online and Face-to-face Psychological Treatment: Comparative Study

**DOI:** 10.2196/36775

**Published:** 2022-05-02

**Authors:** Josep Mercadal Rotger, Victor Cabré

**Affiliations:** 1 Institut Universitari de Salut Mental Vidal i Barraquer Universitat Ramon Llull Barcelona Spain

**Keywords:** online psychological intervention, therapeutic alliance, digital health, mental health, mental health education, mental health treatment, health interventions, health professional, online health, web-based health, intervention modality

## Abstract

**Background:**

Since the COVID-19 pandemic, the number of online mental health treatments have grown exponentially. Additionally, it seems inevitable that this technical resource is here to stay at health centers. However, there is still very little scholarly literature published on this topic, and therefore, the impact of the changes that have had to be dealt with in this regard has not been studied.

**Objective:**

This study aims to evaluate the differences in the establishment of the therapeutic alliance (TA) based on the intervention modality (online or face-to-face), the type of attachment, and diagnosis.

**Methods:**

A total of 291 subjects participated in the study, 149 (51.2%) of whom were men and 142 were (48.8%) women between the ages of 18 and 30 years. The instruments used were sociodemographic data, SOFTA-o (System for Observing Family Therapeutic Alliances—observational), and Relationship Questionnaire.

**Results:**

The results show that the treatments conducted face-to-face obtain significantly better scores in the creation of the TA than those conducted online (*t*=–42.045, *df*=289, *P*<.001). The same holds true with attachment, in that users with secure attachment show a better TA than those with insecure attachment (*t*=6.068, *P*<.001,), although there were no significant differences with the diagnosis (*F*=4.566, *P*=.44), age (*r*=0.02, *P*=.70), and sex (*t*=0.217, *P*=.33).

**Conclusions:**

We believe that professionals are not yet prepared to conduct remote treatment with a degree of efficacy similar to that of face-to-face. It is essential for professionals to receive training in this new technical resource and to understand and incorporate the variants it entails into their daily practice.

## Introduction

### Background

It seems inevitable that online psychological treatments are here to stay in mental health centers and services. The pandemic caused by COVID-19 has accelerated their advent and normalization among mental health professionals, forcing most of their psychotherapeutic activity to shift to the online methodology. Therefore, in a brief period of time, therapists and patients have had to adapt to conditions that forced them to change certain variables, especially the setting, without prior planning or awareness of what other changes they would have to grapple with besides technological ones [1]. Nonetheless, the future of online and face-to-face treatments, once the health crisis is over, is still unclear.

Some authors [2,3] claim that online modalities have facilitated the availability of mental health services during the pandemic. Acero et al [4] further claim that online treatment has facilitated access to mental health services not only in situations caused by COVID-19 but also for people living in rural environments or far from urban nuclei. In this sense, several studies have been published, which conclude that online psychological treatments during the pandemic have led to significant improvements in patients’ concerns with COVID-19 and a significant drop in symptoms such as anxiety, depression, and insomnia [5,6]. However, there is still a lot of room to study the differences between the therapeutic alliance in online and face-to-face psychological treatment in terms of efficacy and quality.

### Online Psychological Interventions

Different authors [7,8] warn that the use of these digital resources is not without consequences in the patient-therapist relationship and that therapists should use these new communicative devices with a great deal of care and knowledge, especially not knowing the risks they could entail for the patient and the therapeutic relation. In fact, some authors agree that there is limited knowledge about the feasibility and acceptability of eHealth interventions in relation to the clinical characteristics of certain types of patients, such as psychotics. The studies by these authors conclude that the level of acceptance among patients with psychosis is high and offer evidence that both online interventions and the use of artificial intelligence can serve as a profitable, accessible, and effective therapeutic agent [9,10].

In some countries such as Brazil, online psychological treatment may only be carried out if the purpose is to research its efficacy [11], with the argument that this new technical resource may have limitations or legal or ethical problems related to its practice. Other countries such as Italy claim that many professionals are not prepared either methodologically or technologically for the change from traditional therapies to digital or online therapies [12]. In another study also conducted in Italy, only 18.3% of the therapists reported having experience with online treatments, and even though 62.6% of the psychologists were in favor of online treatment, they saw many limitations and had many reservations about ethical and legal issues, in addition to technical and methodological ones [13]. In this sense, De la Torre and Pardo [14] do not recommend holding online sessions at times of crisis or under specific conditions such as a lack of emotional control characteristic of people with psychotic disorders, severe depression, or situations of severe violence and abuse, among others, as they must be addressed in a specific way and in some cases by a multidisciplinary team. In fact, in a study conducted in Germany, therapists claim that treatment conducted face-to-face is much more efficacious than online treatment [15].

Rollman et al [16] conducted a study in which they compared the application of online and face-to-face treatment in a sample of 704 patients who had anxiety and depression; they concluded that online therapy did not provide any additional benefit over face-to-face therapy. However, Rathenau et al [17] affirm that the main predictive factor of the efficacy of online treatments is the therapist’s attitude toward it. Other authors claim that live, face-to-face human treatment is not comparable to online treatment, and that while at times it can be a good resource and even a good complement, under no circumstances can it be better and “more real” than face-to-face treatment [7]. In this sense, Knaevelsrud and Mearcker [18] cautioned that we know little about how the therapeutic relationship evolves over the internet and whether it influences the outcome of the treatment, as it does in traditional face-to-face treatments. However, the meta-analysis carried out by Lin et al [19], in which the findings between teletherapy and in-person therapy were compared, concluded that there were no significant differences between teletherapy and face-to-face therapy in the results at posttreatment (*g*=0.043), at follow-up (*g*=0.045), or in attrition rates (rate ratio=1.006). In addition, the within-group findings showed that teletherapy produced a large reduction in symptoms at posttreatment (*g*=1.026) and at follow-up (*g*=1.021). Thus, these findings provide empirical support for the practice of teletherapy, and client outcomes in teletherapy do not differ from in-person versions of treatments.

### Therapeutic Alliance

The TA is one of the most investigated variables related to success in psychological interventions, regardless of the theoretical orientation.

Many authors affirm that the TA is the main predictor variable of results in mental health treatments [20-24].

Bordin [25] proposes that the TA has three components: agreement between therapist and patient about the goals of therapy; agreement on the tasks necessary to achieve those goals; and affective bond between therapist and patient, necessary to withstand the difficulties of therapeutic change. For Muran [26], the TA implies that an intersubjective negotiation between patient and therapist about the needs and desires of the other underlies all treatment. Luborsky et al [27] also made interesting contributions by distinguishing two phases in the development of the TA. At the beginning of treatment, the Type I alliance implies that the patient trusts that the treatment will help, and the therapist offers a warm, supportive, and caring relationship. Both aspects create the conditions for the treatment to start and develop. Later, the Type II alliance is based on joint effort to overcome difficulties and bring about change. This implies trust and commitment on the part of the patient and a solid experience of collaboration with the therapist.

In this sense, there is still no certainty as to whether the establishment of the TA in online interventions is as powerful as in face-to-face interventions. However, a study by Anderson et al [28], in which the differences in the establishment of the TA in adolescents with anxiety were studied, the results showed that the adolescents did not report differences between those who had received face-to-face treatment and those who had received it online. Along the same lines, in a systematic review that evaluated the differences in the establishment of the TA between web-based and face-to-face interventions, it was concluded that the quality of the TA established in web-based interventions is, at least, the same as in face-to-face interventions. In addition, it also indicated that there was a relationship between the TA and the results of the interventions [29]. Flückiger et al [30] conducted a meta-analysis in which they collected 295 independent studies that covered more than 30,000 patients in online and face-to-face treatment. The study investigated the relationship between TA and treatment outcome. The results indicated that a good TA was a predictor of better therapeutic results in both treatments (online and face-to-face). However, the results were significantly better in face-to-face treatments than in web-based treatments. There is also another meta-analysis carried out by Kaiser et al [31], which aimed to summarize the association between TA and outcome in therapist-assisted online interventions. Overall, 51 effect sizes were extracted from 20 included studies. The average weighted effect size is *r*=0.203 (*P*<.001). The correlation was larger when alliance was measured near the end of an intervention. There was no impact of therapist contact frequency or mode and availability of self-help content on the effect size. Therefore, it is concluded that TA and outcome are significantly correlated in web-based therapy. That is, it highlights the importance of a stable alliance in web-based interventions and suggests that fostering the alliance could be beneficial for treatment success.

### Therapeutic Alliance and Attachment

Attachment theory provides a model for understanding development within the context of the child’s primary and formative relationships, on the one hand, and an adult’s orientation toward lifelong intimate connections and social relationships, on the other. Researchers in psychotherapy have linked measures of patient attachment to the therapeutic alliance, therapeutic process, and therapeutic outcomes. The attachment organization and the therapist’s ability to mentalize play an important role in establishing a good therapeutic alliance and, therefore, in therapeutic success [32].

Smith et al [33] conducted a systematic review of research that has examined the relationship between self-reported patterns of attachment and TA. The results suggest that patients who rate themselves as having a more secure attachment pattern are likely to rate the alliance as stronger. The idea is that patients project their internal working models onto the therapist and the therapist-patient relationship, so that the patient’s attachment patterns affect how the two parties interact with each other and thus the formation and the maintenance of their TA [34]. Patients who have a secure attachment are better able to engage in self-exploration, engage in self-disclosure, develop collaborative understanding with the therapist, and be able to reflect on and evaluate their past and current relationships [35]. These skills would help securely attached patients to form a good-quality TA and maintain it by repairing any breaks that develop. Conversely, patients with an insecure attachment pattern may avoid interpersonal closeness with the therapist or worry about the therapist’s investment in them. As a result, this can prevent or delay the formation of a good quality TA [33,36,37].

Daniel [38] advances the idea that therapeutic change occurs when insecure clients, contrary to their previous experience, experience a supportive and responsive relationship with their therapist. If this experience deviates significantly from the individuals’ early prototype model, their central attachment pattern may change. Consistent with this idea, studies have reported that decreases in symptom severity during psychotherapy are associated with increases in self-reported secure attachment [39,40].

This is the context within which we set out to conduct this study, whose main objective is to evaluate the differences in the establishment of the TA in online compared to face-to-face treatments.

Likewise, we shall also evaluate the subjects’ type of attachment and what effects this has on the establishment of the TA.

## Methods

### Participants

A total of 291 subjects participated in this study anonymously and voluntarily, 149 (51.2%) of whom were men and 142 (48.8%) women. The subjects were between the ages of 18 and 30 years, with a mean age of 23.1 (SD 2.82; Table 1).

The participants came to the psychological guidance and consulting service voluntarily and free of charge and were invited to participate in the study. The main objective of this service is to psychologically assess or explore the users from 2 universities in Barcelona, and if needed, to refer them to the corresponding services in the public health care network. Participants who were involved in fewer than 3 sessions were excluded.

### Instruments

The participants responded to the following questionnaires: (1) sociodemographic data—sociodemographic data such as sex, age, whether the treatment was online or face-to-face, and the diagnostic was collected ad hoc; (2) therapeutic alliance—SOFTA-o (System for Observing Family Therapeutic Alliances—observational) for patients [41]; this instrument was created simultaneously in English and Spanish as a transtheoretical tool for research and practice on the TA. In this case, the patient version was used. The measure is based on three dimensions: engagement in the process, emotional connection, and safety. It also provides an overall score. The 12 items, both negative and positive, are related to patients’ behaviors, which are grouped within these 3 dimensions; and (3) attachment—Relationship Questionnaire is a brief self-report that was developed by Bartholomew and Horowitz [42] to evaluate adults’ attachment style based on continuous measures and categorical results. First, the person being evaluated is presented with four prototypical descriptions of the types of attachment in Bartholomew’s model (secure, anxious-preoccupied, dismissive-avoidant, and fearful-avoidant) and is asked to decide with which one they identify the most. Secondly, they are asked to rate their degree of agreement with each of the prototypical definitions of attachment on a 7-point Likert scale [43-47].

### Procedure

All the subjects filled out the SOFTA-o and the Relationship Questionnaire before the exploration began and filled out only the SOFTA-o after it. It is understood that the TA with the therapist will change if the exploration was a positive experience, but the type of attachment will not, as this construct is stable over time.

The explorations lasted between 3 and 5 sessions. The subjects themselves chose whether they wanted to be treated face-to-face or online. The online interventions were carried out through videoconference.

The subjects filled out the questionnaires individually and independently, and they were only assisted by the researcher if they requested help.

### Ethics Approval

The study was approved by Research Ethics Committee of the Vidal i Barraquer Mental Health University Institute.

## Results

### Description of Analyses

The statistical analyses were conducted using SPSS statistical package (version 27.0, SPSS Inc). First, the descriptive results of the sociodemographic data, the TA, attachment, and the diagnosis were presented. Subsequently, the relations between the TA and the intervention modality, attachment, sex, age, and diagnostic were presented. Next, the mixed model analysis was conducted. To do so, an unstructured variance-covariance matrix was calculated via the restricted estimation of maximum likelihood. The TA before and after treatment, treatment modality, attachment scale, and their interactions were considered fixed effects. Finally, gender and age were also included as fixed factors. The random effect was the subjects’ intersection parameter. The degrees of freedom were calculated with the Satterthwaite approximation. The end model was chosen by recalculating the models with and without interaction via maximum likelihood in order to compare the significance of the change on the Akaike information criterion (AIC). The residuals of the prediction and of the random factor were inspected via a quartile-quartile plot to assess the suitability of the model.

### Descriptive Results of the Sociodemographic Data, Therapeutic Alliance, Attachment, Intervention Modality, and Diagnosis

As shown in Table 1, the percentage of men and women was almost similar, with 142 (48.8%) women and 149 (51.2%) men. The mean age was 23.1 (SD 2.82) years; 43.6% (n=127) chose the web-based option while 56.4% (n=164) chose face-to-face. The differences were not significant (*t*=0.210, *df*=289, *P*=.91).

The most prevalent diagnosis was anxiety (n=91, 31.3%), followed by depression (n=45, 15.5%) and grief (n=29, 10%). We can also see that 63.6% (n=185) of the participants had a secure attachment, while 36.4% (n=106) had an insecure attachment. Finally, regarding the TA, we see that prior to the treatment, the mean SOFTA-o score of the subjects was 8.62 while after treatment, it was 36.78.

Comparison between age, sex, modality, diagnosis, and attachment in relation to the therapeutic alliance before and after treatment.

We conducted *t* tests for the variables sex, modality, and attachment; we used the Pearson correlation coefficient for age and TA and ANOVA for the diagnosis.

Via the Pearson correlation coefficient, Table 2 shows significant relations in the scores on the TA at the two times when the questionnaire was administered. We see that between the pre- and postadministrations, there is a correlation of *r*=0.09 and *P*<.001.

If we examine the relationship between TA and age, we see that prior to the treatment, there is a correlation of *r*=–0.10 and *P*=.08, while afterward, it was *r*=0.02 and *P*=.70. Therefore, there are no significant differences in the establishment of a better TA according to age.

As we can also see in Table 2, the *t* test for independent samples revealed that there are no significant differences in the TA prior to the treatment, with the treatment modality (web-based and face-to-face) *t*=0.150, *df*=289, *P*=.88; attachment (secure and insecure) *t*=–0.835, *P*=.39; and sex (male and female) *t*=1.430, *P*=.16. By contrast, after the treatment, we do find significant differences in the treatment modality *t*=–42.045, *P*<.001, and the type of attachment *t*=6.068, *P*<.001, but not sex, *t*=0.217, *P*=.33. Therefore, we can conclude that the face-to-face modality shows significantly better results in terms of establishing a good TA compared to web-based treatments. The same holds true for attachment, where having a secure attachment leads to significant differences in the development of a better TA.

Finally, regarding the diagnosis, we conducted an ANOVA to determine whether there were differences in the establishment of a better TA by diagnosis, and the results both before and after the treatment showed that there are no significant differences (*F*=1.097, *P*=.37 and *F*=4.566, *P*=.44, respectively; degrees of freedom between groups, within groups, and total were 9, 281, and 290, respectively; Tables 2-4).

**Table 1:** Descriptive results.

Characteristics		Values
Age (years), mean (SD; range)		23.1 (2.82; 18-29)
Pre-TA^a^ scores, mean (SD; range)		8.6 (3.03; 3-18)
Post-TA scores, mean (SD; range)		36.8 (13.88; 11-56)
**Gender, n (%)**		
	Male	149 (51.2)
	Female	142 (48.8)
**Modality, n (%)**		
	Web-based	127 (43.6)
	Face-to-face	164 (65.4)
**Attachment, n (%)**		
	Secure	185 (63.6)
	Insecure	106 (36.4)
**Diagnosis, n (%)**		
	Anxiety	91 (31.3)
	Depression	45 (15.5)
	Grief	29 (10)
	Mistreatment	25 (8.6)
	Family problems	16 (5.5)
	Couple problems	16 (5.5)
	Concentration problems	15 (5.2)
	Social relation problems	28 (9.6)
	Adaptation problems	23 (7.9)
	Others	3 (1)

^a^TA: therapeutic alliance.

**Table 2:** Therapeutic alliance and age correlation before and after intervention.

Correlation	Value		
	*r*	*P* value	Age, *r* (*P*) value
TA^a^ before treatment	0.092	<.001	–0.102 (.08)
TA after treatment	0.092	<.001	0.022 (.70)

^a^TA: therapeutic alliance.

**Table 3:** Therapeutic alliance comparison between the groups before and after intervention.

Tests		Values		
		*t*	*df*	*P* value
**TA^a^** before treatment				
	Modality	0.15	268.130	.89
	Attachment	–0.853	203.183	.40
	Gender	1.403	284.221	.16
**TA after treatment**				
	Modality	–42.045	222.357	.001
	Attachment	6.068	217.342	.001
	Gender	0.22	287.029	.33

^a^TA: therapeutic alliance.

**Table 4:** Therapeutic alliance comparison by diagnosis.

ANOVA	Diagnosis		
	Mean square	*F*	*P* value
TA^a^ before treatment	10.084	1.097	.37
TA after treatment	792.356	4.566	.44

^a^TA: therapeutic alliance.

### Analysis of the Mixed Model

In the model without interactions, the pre-post change in the TA was significant (*t*_576.0_=44.020, *P*<.001), as was the treatment modality (*t*_576.0_=18.804, *P*=.72). Age, gender, and attachment did not reach the level of significance (*t*_576.0_=0.492, *P*=.62; *t*_576.0_=0.17, *P*=.87; and *t*_576.0_=1.048, *P*=.30, respectively).

The model with interactions (AIC=3305.5, with 12 parameters) was significantly better (χ^2^_4_=742.78, *P*<.001) than the model without interactions (AIC=4040.3, with 8 parameters). The interaction between the time of the evaluation and the therapeutic modality was highly significant (*t*_287.0_=32.296, *P*<.001). In the web-based treatment, the mean score on the SOFTA rose by 13.5 points (SD 5), while in the face-to-face treatment, it rose 39.6 points (SD 5.1). The interactions between evaluation and attachment and modality and attachment were not significant (*t*_287.0_=1.248, *P*=.21 and *t*_534.3_=0.363, *P*=.72, respectively). In the inspection of the residuals, no gross deviations were found compared to a normal distribution.

## Discussion

### Principal Findings

The results of this study show that the interventions carried out in person, with a sample of subjects aged between 18 and 30 years, obtain significantly better scores in the creation of the TA compared with those carried out with the web-based methodology. The same occurs with attachment, where users with secure attachment establish a better TA compared with those with insecure attachment. In relation to the variables’ diagnosis, age and sex, there were no significant differences.

### Complementary Results (Sample, Diagnosis, and Sociodemographic Data)

First, we should highlight that this is a sample of university students, so we can assume a high sociocultural level with a social network (at least in terms of their belonging to the educational community: teachers, classmates, etc) and a certain predisposition to establish relational bonds (at least with referents in education). Likewise, they belong to an age group with knowledge and skills of the new technologies and therefore have a low level of interference and inconvenience associated with the use of this variable.

In terms of the modality chosen, the members of the sample distributed themselves in a balanced fashion (43.6% web-based and 56.4% face-to-face), with a slight preference for face-to-face treatment. We may think that this may be a pattern that is tending to gain ground in this age group, in a socioeconomic milieu that enables them to have sufficient technological resources and in a metropolitan setting that minimizes the difficulties of access to face-to-face encounters (remote residences, precarious environments, etc). It is likely that based on the experience of the pandemic, these patients’ initiative, at least in initial contacts, includes both methodologies. The fact that there was a slight predominance of those who requested face-to-face treatment seems to reflect the caregiving logic, in which the vast majority of conflicts associated with mental health directly imply other people with whom one has interactions in face-to-face settings (family, friends, partner, etc). In fact, Cabré and Mercadal [8] claimed that live treatment in person is not comparable to web-based treatment, even though at times it may be a good resource or be complementary; however, under no circumstances can it be better and “more real” than face-to-face. Nonetheless, the significant percentage (43.6%) of those who requested to receive treatment online leads us to believe that this choice may be part of certain initial defensive strategies precisely for the same reason.

Knaevelsrud and Mearcker [18] caution that little is known about how the therapeutic relationship evolves in web-based treatments and whether it influences the outcome of treatment, as it does in traditional face-to-face treatments. In this sense, what we still do not know with the results of this study (and this is a limitation) is whether these percentages of the initial choice stay the same when the psychological treatment consolidates; that is, whether once established with a psychotherapist, part of this group that initially preferred treatment via safety or distance measures (web-based) would prefer to see the therapist face-to-face as they feel safer, less threatened, and more trusting of the other and themselves.

In terms of the diagnosis, it is difficult to establish patterns with such a general and unspecific set of symptoms. Nonetheless, it is likely that in symptoms in which clinically active depressive features predominate, the first choice will tend to be the contact that is the “easiest” and entails the least effort, which is apparently the online connection (even though these same clinical components may respond better to closer human contact). In grief (even though it also contains these components of sadness and anhedonia), we may believe that the need to have close contact with the other and receive affection from them, without filters, may push the demand for face-to-face over web-based treatment. Finally, in symptoms in which anxious contact predominates, especially regarding human or relational contact (eg, social phobias, separation anxieties, and persecutory anxieties), the first choice may be heavily conditioned by this experience, defensive strategies will probably predominate, and thus web-based methodologies may be preferred. In fact, 73.2% (n=23) of the subjects in our study with a diagnosis of depression chose the web-based modality, 67.8% (n=20) of those who were grieving chose the face-to-face modality, and 59.6% (n=54) of the subjects who had anxiety preferred the web-based option.

### Attachment and Therapeutic Alliance

The results of our study show that participants with secure attachment developed better TA compared with those with insecure attachment. These results are in line with other research projects in which it is concluded that a secure attachment predicts a better TA [33,35]. In fact, there are also studies that provide the same conclusions from the opposite side: people with insecure attachment have worse TA [36,37]. In our opinion, it is logical that the subjects with secure attachment develop a better TA. Therefore, if we see that the TA consolidates over the course of a few sessions (which comprise the exploration), it is likely that in a new (and hypothetical) choice to continue (via treatment), the percentage of the same patients preferring face-to-face would rise. In fact, Travis et al [39] and Siefert and Hilsenroth [40] state that the decrease in symptoms during therapy is related to the establishment of a more secure attachment. In any case, if the initial choice was not a reflection of this defensive variable posited above, it is likely that the end outcome of the treatment would be similar (in terms of its efficacy) in both modalities. Nor can we discard the possibility of the opposite: if the TA was adequately established in the face-to-face exploration, this “relational footprint” may make it likely that the percentage of patients who (for different practical reasons) request to continue the treatment (or start psychotherapy) using the web-based method (with the same professional with whom they established at a solid TA) would increase, and there would be no reason to think that the outcome, in terms of efficacy, could not be optimal. Regardless, these aspects entail limitations in this study and should continue to be researched in more extensive longitudinal studies.

### Therapeutic Alliance and Modality (Web-Based or Face-to-face)

When we compared at the moment before the intervention if there were differences between TA and modality (web-based or face-to-face), the results showed that there were no differences between these two groups. However, when comparing the modality and the TA at the time after the intervention, the results showed that the face-to-face modality presents significantly better results when establishing a good TA, compared to web-based interventions. These results dispute the conclusions reached by investigations such as that of Anderson et al [28], in which it was concluded that there were no differences between web-based and face-to-face interventions when establishing a good TA.

Finally, we see how the interaction at the time of evaluation and the therapeutic modality were significant; indeed, we found that the score on the TA was 3 times higher in the face-to-face modality (39.6), compared with that in the web-based modality (13.5). Therefore, even though the outcomes may be quite positive in the web-based modality (since it is assumed that TA has improved throughout the intervention), these results are contrary to those reported by Sucala et al [29], who conclude that the quality of TA in web-based interventions is, at least, the same as in face-to-face interventions. However, several studies [20-24,30,31] state that TA improves therapeutic results both in face-to-face and web-based interventions. In fact, Eichenberg [15] stated that face-to-face is more effective than the web-based modality and therefore should be used whenever possible.

Furthermore, we believe that is it obvious, as Tullio et al [12] noted, that professionals are not yet prepared to conduct remote treatment with a degree of efficacy similar to that of face-to-face. Furthermore, only 18.3% of therapists reported having experience with web-based interventions, although 62.6% are in favor of them [13].

For all these reasons, we believe that, as Mercadal and Cabré [1] stated, it is essential for professionals to receive training in this new technical resource and to understand and incorporate the variants it entails into their daily practice.

## Abbreviations

AIC: Akaike information criterion
SOFTA-o: System for Observing Family Therapeutic Alliances—observational
TA: therapeutic alliance
